# Establishment and characterization of a new and stable collagen-binding assay for the assessment of von Willebrand factor activity

**DOI:** 10.1111/ijlh.12019

**Published:** 2012-10-29

**Authors:** Y Ni, J Nesrallah, M Agnew, F J Geske, E J Favaloro

**Affiliations:** * Precision BioLogicDartmouth, NS, Canada; † Institute of Clinical Pathology and Medical Research, Westmead HospitalWestmead, NSW, Australia

**Keywords:** Collagen binding, von Willebrand factor, von Willebrand disease, ristocetin cofactor, ELISA

## Abstract

**Introduction:**

Laboratory diagnosis of von Willebrand disease (VWD) requires determination of both von Willebrand factor (VWF) protein levels and activity. Current VWF activity tests include the ristocetin cofactor assay and the collagen-binding assay (VWF:CB). The goal of this investigation is to characterize a new collagen-binding assay and to determine its effectiveness in identifying VWD.

**Methods:**

Analytical studies were carried out to characterize the performance of a new VWF:CB ELISA. Additionally, samples from a normal population were tested as were well-characterized type 1 and type 2 VWD samples.

**Results:**

Repeatability and within-laboratory precision studies resulted in coefficients of variation (CVs) of ≤11%. A linear range of 1–354% (0.01–3.54 IU/mL) was determined, along with a limit of detection and a lower limit of quantitation of 1.6% and 4.0% (0.016 and 0.04 IU/mL), respectively. Samples tested from apparently healthy individuals resulted in a normal range of 54–217% (0.54–2.17 IU/mL). Known VWD type 1 and type 2 samples were also analyzed by the ELISA, with 99% of samples having VWF:CB below the normal reference range and an estimated 96% sensitivity and 87% specificity using a VWF collagen-binding/antigen cutoff ratio of 0.50.

**Conclusion:**

This new VWF:CB ELISA provides an accurate measure of collagen-binding activity that aids in the diagnosis and differentiation of type 1 from type 2 VWD.

## Introduction

Von Willebrand disease (VWD) is considered the most common inherited bleeding disorder, with conservative estimates of incidence in the general population of around 100 per million persons 1 and up to one in 1000 in the primary care setting 2. There are currently six types of defined VWD, as caused by a variety of mutations 3–7. Types 1 and 3 are quantitative defects in von Willebrand factor (VWF), with type 1 having reduced levels of functionally normal VWF and type 3 showing virtually absent VWF. Type 2 VWD defines qualitative defects that typically result in low-to-normal levels of VWF but reduced or absent VWF functionality. Type 2 VWD comprises four distinct types: 2A, 2B, 2M, and 2N. Type 2A VWD is characterized by absence of intermediate and high molecular weight (HMW) VWF multimers. Type 2B is characterized by increased affinity of VWF to platelets resulting in a loss of HMW multimers. Type 2M VWD demonstrates decreased VWF-dependent platelet adhesion but does not have a deficiency of HMW multimers. Finally, type 2N is classified by reduced binding affinity of VWF to Factor VIII (FVIII) 6,7.

Diagnosis of VWD is made in individuals with personal and family histories of mucocutaneous bleeding and confirmed with laboratory testing. As there are different types of VWD, no single test is sufficient for diagnosis, and testing of both VWF antigen and function must be performed. Using any of the various assays in isolation increases the risk of misdiagnosis 8. VWF protein (antigen; VWF:Ag) levels are determined by enzyme-linked immunosorbent assay (ELISA) or immunoturbidimetric methods. The ratio of VWF activity to antigen is useful in determining VWD subtypes 10. FVIII functional testing (FVIII:C), usually by clot-based assays, can aid in diagnosing VWD, as VWF binds FVIII *in vivo* and acts as a carrier protein. Additionally, multimer analysis, as it provides visualization of the VWF multimers, aids in the classification of VWD, particularly type 2 4.

VWF activity assays include the ristocetin cofactor assay (VWF:RCo) and the collagen-binding assay (VWF:CB), as well as a number of new assays in development 11. The VWF:RCo assay was the first activity assay introduced commercially and is currently the most common method of measuring VWF activity. Ristocetin, an antibiotic, induces VWF-mediated agglutination of fresh or formalin-fixed platelets. In this assay, levels of agglutination with patient samples are compared to a reference plasma sample containing known VWF:RCo activity 4. The VWF:RCo assay has significant limitations. High variability in the VWF:RCo assay has been reported as well as low sensitivity 12 and a lower limit of detection of approximately 20% VWF in many laboratories 13. In addition, a not uncommon mutation in the A1 domain of the VWF gene in certain ethnic groups results in decreased VWF:RCo, even though patients may lack bleeding symptoms and other tests are commonly found to be normal 14.

In part due to the limitations of the VWF:RCo assay, a number of investigators have evaluated the diagnostic capabilities of the VWF:CB 11,15–23. The ELISA-based VWF:CB assay uses collagen (usually type I or III alone or in combination) immobilized on a microplate. Diluted patient samples are added and incubated, followed by detection of bound VWF by addition of an anti-VWF conjugate. A substrate is added, then quantitation is performed on a plate reader, and values are obtained based on a reference curve. The use of the VWF:CB assay has resulted in a substantial reduction (upwards of 50%) in the diagnostic error rate when used in addition to the VWF:RCo assay 9,13. Furthermore, the VWF:CB to VWF:Ag ratio was more specific for the identification of samples with a type 2 multimer pattern than the VWF:RCo to VWF:Ag ratio 8. The VWF:CB assay therefore could result in a significant reduction in the number of multimer assays required to characterize the type of VWD which is advantageous because multimer analysis is time-consuming, expensive, and only performed in a few laboratories 8. A recent study by the North American Specialized Coagulation Laboratory Association (NASCOLA; 24 identified a high error rate by participants performing multimer analysis, such that 5% of laboratories reported false loss of HMW VWF multimers in normal samples, 18% reported false loss of high- or medium- and HMW multimers in type 1 VWD samples, and 22% reported a false normal multimer pattern in type 2A VWD samples. In this same study, type 2 VWD samples were correctly identified by all laboratories using VWF:CB to VWF:Ag ratios but by only one-third of laboratories using VWF:RCo to VWF:Ag ratios.

Recently, six different commercial VWF:CB assays were characterized 17. As a follow-up to this study, the current report further describes one of those assays (ELISA*check*™ Collagen Binding Assay; Precision BioLogic, Dartmouth, Canada) which specifically was identified to yield more favorable discrimination of type 1 and 2 VWD. In the current report, analytical parameters such as precision, limit of detection and quantitation, and linear range are presented, as well as an analysis of a large number of normal and well-characterized type 1 and type 2 VWD samples. The results reconfirm that this new assay is a valuable aid in the detection of VWD and the discrimination between type 1 and type 2 VWD.

## Materials and methods

### Reagents

Reagents used for plate coating include the ELISA Wash Buffer (EWB; 1× = 0.075M NaCl, 0.005M Trizma pH 9.1, 0.05% Tween-20, 0.05% Proclin 950); bromocresol purple solution (1% in 0.2M NaHCO_3_, 0.1% NaN_3_); coating buffer (10 mm acetic acid, 0.01% bromocresol purple solution); stabilizing buffer (1% BSA, 5% sucrose in 1× EWB); and blocking buffer (0.2M NaHCO_3_, 1% BSA, 0.001% bromocresol purple solution, 0.1% NaN_3_). The assay diluent contains 1% BSA in 1× PBS (Sigma #P5368), 0.1% Tween-20, 0.02% thimerosol, and 0.001% bromocresol purple solution.

Sodium chloride and acetic acid were obtained from Fisher Scientific (Whitby, Ontario, Canada). All other of the above-mentioned chemicals were obtained from Sigma-Aldrich (Oakville, Ontario, Canada).

### Preparation of plates

Note: all glassware used in the handling of collagen (graduated cylinders, beakers, etc) were precoated with 1% BSA.

Nunc Maxisorb C8 Lock-well plates (Fisher Scientific) were coated with human type III collagen at 0.7 μg/mL (obtained from Precision BioLogic) in coating buffer, 100 μL/well. Plates were incubated overnight (12–18 h) at room temperature (18–25 °C). The collagen coating solution was decanted, and 150 μL/well of blocking buffer was added to each well and allowed to incubate for at least 60 min at room temperature. Plates were washed with 350 μL of wash buffer with a plate washer (96-well AquaMax 2000; Molecular Devices, Sunnyvale, CA, USA), twice with a 30-s soak. 150 μL of stabilizing buffer was added to each well and incubated at room temperature at least 30 min. The stabilizing buffer was then aspirated, and plates were air-dried at room temperature for 16–30 h. Plates were stored in a vacuum-sealed pouch with desiccant at 4 °C until use.

### Preparation of detector conjugate

HRP-conjugated goat anti-human VWF antibody (made by Precision BioLogic) was diluted 1 : 200 in assay diluent 10 min prior to use.

### Collagen-binding ELISA

All reagents (except the detector conjugate) were warmed to room temperature before running the assay. A five-point calibration curve was prepared by threefold serial dilution in assay diluent using a reference solution prepared from pooled normal plasma. The reference solution value was assigned using the SSC/ISTH Secondary Coagulation Standard, Lot #3. Controls and sample plasmas were diluted 1 : 100 in assay diluent. Calibrators, controls, and samples were added (100 μL/well) to duplicate wells of the collagen-coated plate. 100 μL of assay diluent was added to duplicate wells and used as a blank. The plate was covered with a plate sealer or cover and incubated for 60 min at room temperature. After the incubation, the plate was washed with 1× EWB four times with a 30-s soak between each wash. The diluted detector conjugate was then added (100 μL/well), and the plate was covered and incubated for 60 min at room temperature. The plate was then rewashed, and 100 μL/well of TMB substrate was added (Colorburst Blue TMB/peroxide solution; obtained from ALerCHEK, Inc., Springvale, ME, USA), and the plate was incubated for 10 min at room temperature. 100 μL/well of 1N HCl (Fisher Scientific) was then added to each well, and absorbance was read at 450 nm with a 650-nm reference on a plate reader (Versamax; Molecular Devices). Values of controls and samples were obtained through extrapolation from the reference curve.

### Patient samples

All testing was performed on thawed plasma collected into 3.2% sodium citrate according to standard laboratory procedures at Esoterix, Inc., Englewood, CO, USA 25. 242 plasma samples from apparently healthy individuals were evaluated to determine the normal reference interval. These individuals all had normal prothrombin times (PT) and normal activated partial thromboplastin times (aPTT). 52 type I and 49 type 2 VWD samples that were characterized according to established laboratory procedures and according to manufacturer's instructions (VWF:Ag (STA-Liatest VWF:Ag; Stago, Parsippany, NJ, USA, measured on the Siemens BCS analyzer), VWF:RCo (Siemens Healthcare Diagnostics BC von Willebrand Reagent, measured on the Siemens BCS analyzer), and multimer analysis (Esoterix)) were de-identified and evaluated. Type 2 VWD samples included 18 type 2A, 24 type 2B, and 7 type 2 not otherwise classified based on a VWF:RCo to VWF:Ag ratio of <0.5. De-identified samples used in this study followed IRB approval through Copernicus Group IRB, Protocol #CBA-2009, tracking #LAB1-09-422. IRB approval date was November, 12, 2009 and expiration date November, 11, 2010.

### ELISA analytical parameters

Precision analysis was performed using three plasma samples (cryo*check* Reference Control Normal, cryo*check* Abnormal 1 Reference Control, and cryo*check* Abnormal 2 Reference Control; Precision BioLogic) with different levels of VWF:CB activity and tested in duplicate over 20 days for a total of 40 measurements per plasma sample. The percent coefficient of variation (%CV) was calculated according to CLSI Guideline EP05-A2 26. Within-laboratory precision is defined by Guideline EP05-A2 as total precision within the same facility. To determine the limit of detection (LoD) and the lower limit of quantitation (LoQ), plasma samples with low levels of VWF:CB activity were tested by two operators across three assay lots for a total of 132 measurements. The LoD and LoQ were determined according to CLSI Guideline EP17-A 27. Linearity was determined using a plasma sample with low VWF:CB activity mixed with a plasma sample with high VWF:CB activity. Each sample was tested in triplicate, and linearity was evaluated according to CLSI Guideline EP06-A 28.

## Results

Results of precision studies (repeatability and within-laboratory precision) are shown in Table 1, and identify that all CV's were below 10% for repeatability and ≤11% when measuring within-laboratory precision. The LoD and LoQ were identified as 1.6% (0.016 IU/mL) and 4.0% (0.04 IU/mL), respectively, and the linear range of the assay was determined to be 1–354% (0.01–3.54 IU/mL) (not shown). The normal reference range for VWF:CB activity yielded a mean value of 113% (1.13 IU/mL) with a 2.5–97.5 percentile reference interval of 54–217% (0.54–2.17 IU/mL; Table 2). Values for men and women were similar.

**Table 1:** Precision analysis using three plasma samples tested in duplicate over 20 days for a total of 40 measurements per sample

Sample #	Mean VWF:CB activity% (IU/mL)	Repeatability as %CV	Within-laboratory precision as %CV
1	111 (1.11)	7.8	10.8
2	37 (0.37)	7.0	9.6
3	11 (0.11)	9.2	11.0

**Table 2:** The normal reference range determined from 242 apparently healthy individual

	VWF:CB activity%(IU/mL)		
	
	Total population	Male	Female
*N*	242	181	61
Mean	113 (1.13)	113 (1.13)	115(1.15)
Median	104 (1.04)	103 (1.03)	105 (1.05)
2.5–97.5 Percentile reference interval	54-217 (0.54–2.17)	53-269 (0.53–2.69)	56-201 (0.56–2.01)

**Figure 1:**
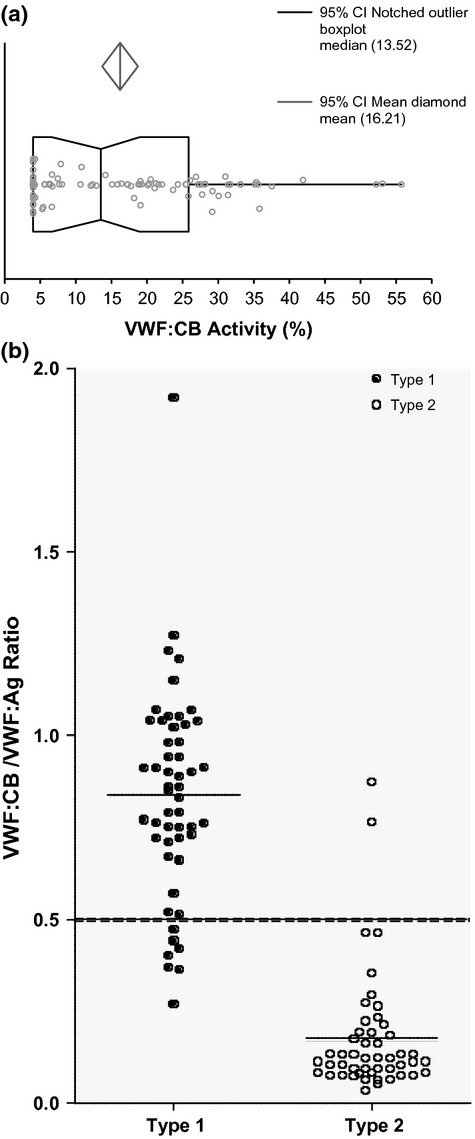
VWD sample analysis. (a) 101 known VWD samples were run on the VWF:CB ELISA and percent collagen-binding activity was plotted. The median (vertical line) and 95% intervals (box) are shown, as well as the mean confidence intervals (diamond shape, above). (b) Type 1 VWD samples are shown as solid black circles, and type 2 VWD samples are shown as open white circles. The lines in each population represent the mean ratio values of each VWD type. The dashed line represents the VWF:CB/VWF:Ag ratio cutoff (0.5) used to differentiate type 1 from type 2 VWD.

The analysis of VWD samples is shown in Figure 1. 100 of 101 (99%) of known VWD samples had a VWF:CB activity below the lower limit of the normal reference interval (Figure 1a). Thus, the assay was able to differentiate VWD samples from normal samples. To further analyze type 1 *vs*. type 2 VWD, VWF:Ag levels were determined for each sample and VWF:CB to VWF:Ag ratios calculated (Figure 1b). Using a cutoff ratio of 0.5 established by standard ROC analysis (Table 3), type 1 and type 2 VWD samples were clearly identified, with an estimated sensitivity to type 2 (from type 1) of 95.9% and a specificity of 86.5% (Table 4).

**Table 3:** Sensitivity/specificity analysis at different cutoff values

Cutoff (VWF:CB/VWF:Ag)	Sensitivity (%)	Specificity (%)
0.4	92	92
0.5	96	87
0.6	96	81

**Table 4:** Collagen-binding activity test results as compared to established laboratory diagnosis

Using a ratio of 0.50 as a cut-off *N* = 101	Established laboratory diagnosis	

	Type 2 VWD	Type 1 VWD
Collagen-binding activity test results		
Type 2 VWD	47	7
Type 1 VWD	2	45

Sensitivity and specificity were determined to be 96% and 87%, respectively

## Discussion

In the current study, we have further evaluated one of the best performing VWF:CB assays in terms of type 1 *vs*. type 2 VWD discrimination 17 to a level of detail not previously provided for a VWF:CB assay. This study was designed to evaluate this new collagen-binding ELISA to establish both analytical and clinical assay parameters and to determine its usefulness in aiding in the diagnosis of VWD. The assay demonstrated good precision (repeatability and within-laboratory precision) with a linear range of 1–354% (0.01–3.54 IU/mL) and a lower limit of quantitation to 4.0% (0.04 IU/mL). The normal reference range was 54–217% (0.54–2.17 IU/mL). Additionally, the VWF:CB assay was very effective in differentiating VWD samples from healthy samples. Finally, a comparison of known type 1 and type 2 VWD samples was undertaken using the VWF:CB assay and VWF:Ag levels. Standard ROC analysis suggested a cutoff of 0.5. Using this VWF:CB to VWF:Ag ratio cutoff, good discrimination between the two subtypes was seen, further suggesting that the assay can be used to distinguish type 1 from type 2 VWD.

VWF binds to exposed collagen upon damage to the vascular surface, resulting in an activation of VWF, which then enables platelet glycoprotein Ib/V/IX recognition (reviewed in 29). VWF binds to collagen through its A3 domain, while the VWF A1 domain is involved in ristocetin-induced platelet activation 30. Therefore, although both VWF:RCo and VWF:CB assays assess VWF activity, they do so through different mechanisms. Due to superior performance of the VWF:CB assay (lower assay variability, lower limit of detection, and better sensitivity to loss of HMW multimers), it has been suggested that the VWF:CB and VWF:Ag combination would be preferable to the VWF:RCo and VWF:Ag combination for those laboratories that want to perform only one VWF functional assay 31. The VWF:CB to VWF:Ag ratio was found to be more accurate in terms of identifying type 2 VWD than the VWF:RCo to VWF:Ag ratio in a recent NASCOLA report and in another published study by Adcock *et al*. 8,24. Addition of the VWF:CB assay also improved diagnostic accuracy in an Australasian-based quality study 9. In this study, laboratories using a VWF:RCo assay without the VWF:CB assay were seven times more likely to misidentify type 1 VWD as type 2. Similarly, laboratories were three times more likely to misidentify type 2 as type 1 or type 3 and four times more likely to misidentify normal patients as having VWD if they used the VWF:RCo assay in the absence of the VWF:CB assay. Ultimately, thorough VWD testing should include measurement of VWF levels (VWF:Ag) and determination of each aspect of function: Specifically, the interaction with platelet glycoprotein 1b (VWF:RCo), as well as subendothelium-collagen interactions (VWF:CB), evaluation of FVIII activity, and in some circumstances multimer analysis 32.

In conclusion, proper identification and typing of VWD requires a panel of assays to allow the best patient care and therapeutic management 33. The VWF:CB assay characterized in this study is a valuable addition to the current VWD diagnostic toolbox.
